# The usefulness of cone beam computed tomography according to age in cleft lip and palate

**DOI:** 10.25122/jml-2022-0209

**Published:** 2022-09

**Authors:** Cristian Dinu, Oana Almășan, Mihaela Hedeșiu, Gabriel Armencea, Grigore Băciuț, Simion Bran, Daiana Opriș, Sergiu Văcăraș, Vlad Iștoan, Mihaela Băciuț

**Affiliations:** 1 Department of Maxillofacial Surgery and Implantology, Iuliu Haţieganu University of Medicine and Pharmacy, Cluj-Napoca, Romania; 2 Department of Prosthetic Dentistry and Dental Materials, Iuliu Haţieganu University of Medicine and Pharmacy, Cluj-Napoca, Romania

**Keywords:** cleft lip and palate, child, cone beam computed tomography, 2D – Two-dimensional, 3D – Three-dimensional, CBCT – Cone beam computed tomography, CLP – Cleft lip and palate, FOV – Field of view, kV – Kilovolt, mAs – Milliampere-seconds, RME – Rapid maxillary expansion, SD – Standard deviation

## Abstract

The current study aimed to evaluate the usefulness of cone beam computed tomography (CBCT) examination in cleft children and adolescents, the age of the first CBCT exposure, and the criteria that justified the first CBCT exposure. A number of 229 non-syndromic cleft subjects aged between 0–22 years receiving treatment in the same specialized surgical center in orofacial cleft treatment were studied. A cleft group of 64 cleft lip and palate (CLP) children with at least one CBCT exposure was identified based on CBCT records. Parameters related to diagnosis and treatment planning in cleft deformity were considered dental anomalies and bone morphology changes. The examiners assessed whether the treatment option was necessary, not necessary, or could not be evaluated at the age of CBCT exposure. A number of thirty-nine unilateral clefts and fifteen bilateral clefts were identified. Posterior palatal cleft was present in six children (mean age: 15.03±5.55 years; male/female ratio: 1/0.8). Alveolar bone morphology changes were found in 90.58% of cases; jaw relationship changes in 71.82%; nasal fossa morphology changes in 74.99%; airway obstruction in 49.98% and maxillary asymmetry in 87.48%. Orthodontic planning was necessary for 85.93% of CLP patients, and orthognathic surgery in 39.05%. The usefulness of CBCT in patients with CLP varied with age, with reduced value for the evaluation of impaction and root resorption under the age of ten.

## INTRODUCTION

Cleft lip and palate (CLP) represent a complex malformation that requires multidisciplinary treatment. Its therapy extends over the entire growth period and could begin immediately after birth. The clinical manifestation of CLP children includes midfacial growth deficiency associated with the retruded maxilla, facial asymmetry, maxillary constriction, or morphological changes in the nasal cavity [1], and velopharyngeal insufficiency [2]. These abnormalities are mainly related to the congenital defect itself, but the surgical scars could also lead to facial deformities [3]. Prenatal biomarkers for non-syndromic cleft lip and palate using clusters have been described [4]. The diagnosis and treatment of the facial anomalies associated with a cleft should form the basis of an interdisciplinary approach, radiologic investigation bearing an important contribution. In patients with unilateral cleft lip and palate, CBCT is employed to evaluate the asymmetry of the lower jaw, face, and palate [5].

The clinical examination in a specialized center for craniofacial deformities should be performed early after birth, and comprehensive coaching of the parents by a multidisciplinary team will aim to outline the main protocols for diagnosis and treatment. The radiological examination can be recommended in different stages of child development for monitoring the dental and bone growth, as well as in different stages of the cleft on behalf of the surgical or orthodontic treatment. Therefore, the CBCT effectiveness or radiation risks should be considered related to its benefits for treatment protocols applied in CLP children.

The use of CBCT in cleft patients could start in the first years of life when it represents an aid in diagnosis. A customized acrylic palate plate can be manufactured starting at a very early age, to prevent tongue insertion in the cleft. Around the age of six months, lip repair comprising nose and vestibule reconstruction is performed, followed by the closure of the hard and soft palate at 18 months. After two years, the treatment protocol is orientated towards speech therapy, hearing function improvement, and monitoring of dental eruption. The radiological examination during the temporary dentition is necessary only in cases of dental or periapical pathology [6].

In childhood, CBCT examination is used for tooth assessment during mixed and permanent dentition. Orthodontic treatment is a major part of cleft therapy. This therapy is intensified during the mixed and permanent dentition. Prosthetic rehabilitation of the missing maxillary incisors or gap closure with orthodontics can be performed [7]. Conservative dental treatments complete the multidisciplinary management of clefts at all ages, as numerous dental pathologic conditions accompany the malformation: impacted teeth, supernumerary teeth, root resorption, and hypodontia [8, 9].

During the age of nine-twelve years, alveolus grafting (secondary osteoplasty) is performed. The three-dimensional (3D) CBCT evaluation of the bone defect volume is needed [10], and technical details for graft procedures could be easily obtained from the CBCT images [11].

During the teenage years, CBCT is useful for establishing the possibility or limits of orthodontic tooth movements, as well as for evaluating the thickness of the cortical bone. Both orthodontic and dental treatments require proper diagnosis and are based on image evaluation [12]. Owing to the complexity of the local conditions and the need to assess the deformity in the three planes of space, as well as in the treatment planning according to the provisioned future development, CBCT becomes the required imaging modality to offer the necessary details [13].

After the bone growth is completed, CBCT is used for orthognathic surgery planning and results assessment. Thus, CBCT becomes the most important tool in the diagnostic work-up. It is used to assess the position and relationship between the jaws, but also the volume of the pharyngeal airway [14, 15], the facial aspect, and/or asymmetry [16].

When the development of the maxilla is affected by the cleft scars, a transversal maxillary deficit can appear, which affects the occlusion and the facial aspect. This deficit can be assessed with CBCT, and an indication for rapid maxillary expansion (RME) can be made. The results and stability in time can also be assessed by CBCT [17].

The nasal morphological changes, including nasal septum deviation or turbinate hypertrophy, are frequently associated with a decrease in the lower airway volume of the nasal cavity and an increased risk of airway obstruction [18, 19]. CBCT can assess the volume and shape of the nasal cavities and the position of the septum or the turbinates [15]. The airway volume measurement on CBCT could predict the complication of airway obstruction and sleep apnea. CBCT seems to be a simple and effective method for accurately analyzing the airway [20].

The timing for CBCT exposure in cleft children should be related to the treatment procedures [21]. Although CBCT is a common examination for cleft patients, no timing or standardized examination protocol for CBCT in different ages and treatment period was established.

Therefore, in cleft children, a standardized examination protocol that indicates the most appropriate age for the first CBCT examination and that covers every step of the treatment using an optimal number of exposures is needed.

The current study aimed to retrospectively evaluate the usefulness of CBCT examination in cleft children, the age of the first CBCT exposure and the factors that can justify the CBCT examination.

## MATERIAL AND METHODS

A retrospective, cross-sectional, single-center study was performed. A number of 229 non-syndromic cleft children aged between 0–22 years, receiving treatment in the same surgical center specialized in orofacial cleft treatment during 2015–2016, were studied. The inclusion criteria were CLP children or adolescents, aged 0–22 years, with at least an available CBCT record and signed informed consent (either by them or by their placeholders) to participate in the study. The exclusion criteria were CLP adults, or CLP children or adolescents without CBCT images, or patients with craniomaxillofacial syndromes. Probability sampling technique was applied. A CLP group of 64 CLP children and adolescents with at least one CBCT exposure in their lives was identified according to the inclusion and exclusion criteria.

The group of CLP subjects was exposed to two different CBCT machines: NewTom3G (QR, Italy) or/and Promax3D Max (Planmeca, Finland). The number of CBCT examinations throughout their life, the age at examination, and exposure protocol (CBCT unit, milliampere-seconds (mAs), kilovolt (kV), field of view (FOV), and voxel size were noted for each subject.

The first CBCT exposure performed at the youngest age of each child was selected and independently assessed by three examiners (one orthodontist and two maxillofacial surgeons) with a minimum of ten years of experience. The lateral cephalometric measurements, according to Steiner analyses based on CBCT images, were performed by an orthodontist using the Dolphin software (Patterson technology, USA). The results of the 3D cephalometric analyses were provided to the three examiners together with the CBCT images.

Parameters related to diagnosis and treatment planning in cleft deformity were considered dental anomalies and bone morphology changes. The dental anomalies comprised: hypodontia, supernumerary teeth, impacted teeth, and root resorption of the permanent teeth. The examiners recorded their answers for dental anomaly being (1) present, (2) absent, or (3) could not be evaluated at the age of CBCT examination.

The bone morphology was evaluated on CBCT, and the following parameters were assessed: alveolar boundary condition changes as alveolar bone width deficiency and the lingual or buccal bone loss, maxillary asymmetry, jaw relationship changes, and morphological changes in the nasal fossa. The changes in jaw relationship in the vertical and horizontal plane and maxillary asymmetry were evaluated based on CBCT images and 3D cephalometric measurements. Morphological changes of the nasal fossa comprised the floor of the nasal fossa bone defects, asymmetries of the nasal concha or nasal septum. The following possibilities were considered for each parameter: (1) present changes, (2) absent changes and (3) changes that could not be evaluated at the age of CBCT examination.

The available treatment options for cleft patients were: osteoplasty, orthodontic treatment only, orthognathic surgery, and rapid maxillary expansion. The examiners assessed whether the treatment option was (1) necessary, (2) not necessary (3) could not be evaluated at the age of CBCT.

Statistical analysis was performed using R statistics. Counts and percentages of categorical variables were computed. Means and standard deviations (SD) of all continuous variables were calculated. Nonparametric tests were performed to assess the differences between variables. A significance level of p-values below 0.05 was considered statistically significant.

## RESULTS

A number of thirty-nine unilateral and fifteen bilateral CLP patients were identified. Posterior palatal cleft was present in a number of six children (mean age: 15.03±SD 5.55 years; male/female ratio was 1/0.8).

**Figure 1:**
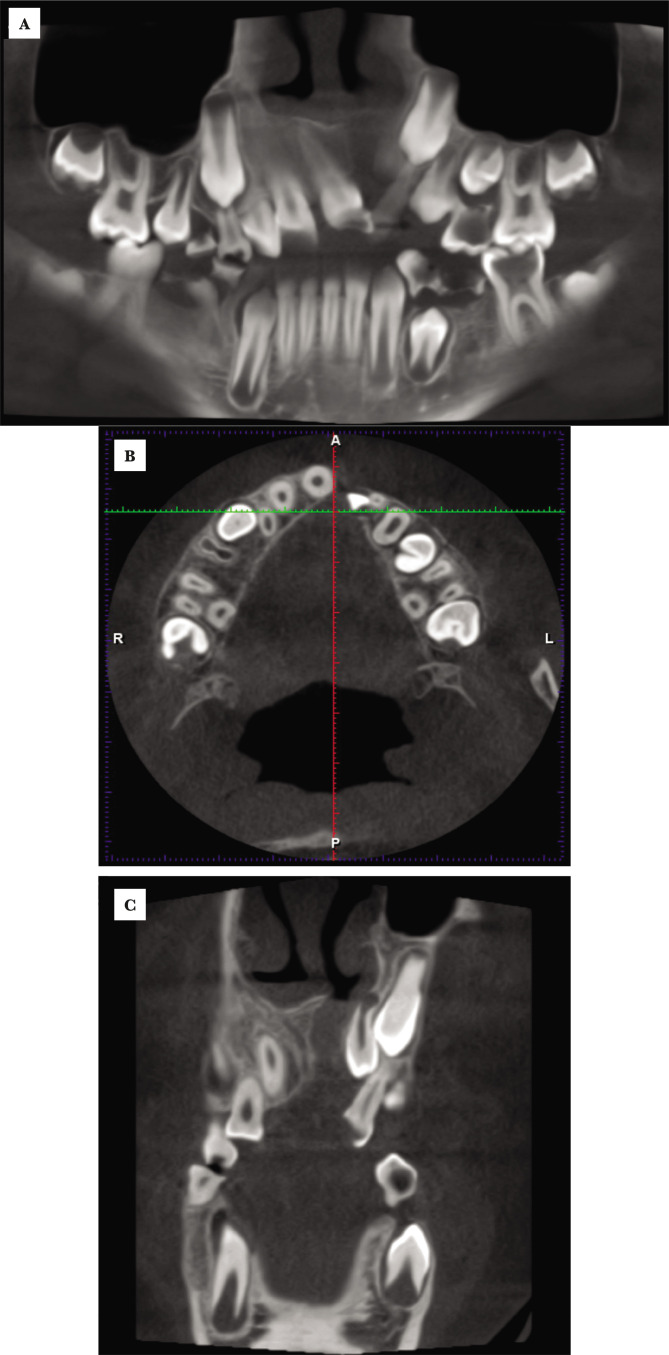
A – Panoramic reformatted image on CBCT examination shows a left CLP with dental anomalies: hypodontia with the absence of first upper right premolar, impacted upper canines with a follicular cyst at the right upper canine and lateral left incisor in the cleft area; mesioversion of the upper lateral right incisor. B – axial CBCT image shows the absence of the upper second right premolar and the position of the teeth in the maxillary jaw, with midline shift; the crown of the lateral left incisor is prominent in the cleft area (A-anterior; P-posterior; R-right; L-left). C – coronal CBCT image shows the dimension of bone defect and the teeth position related to the palate cleft.

The prevalence of dental anomalies in CLP subjects is presented in Table 1. The dental impaction could not be evaluated in 34.37% and the root resorption in 18.75% of cases between 0–10 years. The most frequently encountered dental anomalies were cleft teeth involvement (84.37%), followed by hypodontia (57.81%) and tooth impaction (29.68%). Dental anomalies like hypodontia with the absence of first upper right premolar, impacted upper canines and the dimension of bone defect and the teeth position related to the palate cleft are shown in Figure 1 A–C.

**Table 1:** Prevalence of dental anomalies at the first CBCT examination in CLP children.

Age (years) at first CBCT examination	Impaction (%)	Root resorption (%)	Supernumerary teeth (%)	Hypodontia (%)	Cleft tooth involvement (%)	Number of CLP children
0–5 years	0.00	0.00	1.56	1.56	7.81	5
6–10 years	6.25	0.00	1.56	18.75	20.31	17
11–15 years	7.81	3.12	3.12	9.37	20.31	15
15–20 years	10.93	1.56	1.56	15.62	23.43	17
>20 years	4.68	3.12	0.00	12.50	12.50	10
Total (%)	29.68	7.81	7.81	57.81	84.37	64

The prevalence of morphological changes in cleft children is shown in Table 2. In a percent of 14.06% of children aged below eleven years, the jaw relationship could not be evaluated.

**Table 2:** Prevalence of morphological changes in cleft children.

Age at first CBCT examination	Alveolar bone morphology changes (%)	Jaw relationship changes (%)	Nasal fossa morphology changes (%)	Airway obstruction (%)	Maxillary asymmetry (%)
0–5 years	6.25	0.00	6.25	3.12	4.68
6–10 years	23.43	18.75	17.18	12.5	23.43
11–15 years	20.30	15.6	17.18	9.37	18.75
15–20 years	25.00	21.87	21.87	14.06	25.00
>20 years	15.60	15.60	12.51	10.93	15.62
Total (%)	90.58	71.82	74.99	49.98	87.48

Orthodontic planning was necessary for 85.93% of patients with cleft palate (Table 3). In 32.81% of cases, the evaluation of the graft volume was not possible before eleven years of age. The orthodontic treatment need could not be evaluated in 7.81% of cases on CBCT performed before the age of five and orthognathic surgery in 42.87% of cases aged below twenty years. The examiners could not evaluate the need for RME on CBCT images of children below eleven years in 25% of cases.

**Table 3:** The usefulness of CBCT examination for treatment planning.

Age at first CBCT examination	Graft volume estimation (%)	Orthodontic treatment planning (%)	Orthognathic surgery (%)	Rapid maxillary expansion (RME) (%)
0–5 years	0.00	0.00	0.00	0.00
6–10 years	0.00	25.00	0.00	4.68
11–15 years	14.06	18.75	7.81	14.06
15–20 years	23.43	26.56	15.62	14.06
>20 years	15.62	15.62	15.62	12.5
Total (%)	53.12	85.93	39.05	45.3

## DISCUSSION

The CBCT examination reveals the 3D anatomy of jaws better than the two-dimensional (2D) radiological examination [9, 12, 22]. The usefulness of CBCT in different stages of diagnostic and treatment of cleft anomalies has been reported [23]. The CBCT examination was recommended with a quite constant frequency in all ages of cleft children, except the ages below six years. The results could be suggestive of the fact that no standardized protocol for CBCT examination related to children's age was followed. Moreover, the CBCT irradiation was started at various ages, without significant differences between the age groups. However, the highest number of CBCT exposures was recommended between eleven to fifteen years. Our group has intensely researched on the effects of diagnostic low-dose irradiation and methods to assess the induced changes [24–27]. A more detailed reporting is needed for estimations and for comparing the effective doses of various CBCT units and scanning protocols [23].

The high prevalence of dental anomalies in oral cleft was revealed by many studies [28], with a common genetic origin of cleft and dental anomalies also being suggested [29]. Moreover, the presence of dental anomalies could be suggestive of the oral cleft sub-phenotype [30].

In our study, dental anomalies were encountered in almost the majority of cleft children, CBCT being useful in identifying tooth impaction, root resorption, supernumerary teeth, or hypodontia. Also, CBCT showed the relationship of the teeth with the cleft region, an important number of teeth being related to the bone defect. The CBCT could identify not only the presence and type of the anomaly, but tooth position, as well root development; the dental biotype being better observed on CBCT images than on 2D dental radiographs [31]. However, our study revealed that before the age of ten, there were cases in which CBCT could not predict impaction or root resorption.

The alveolar boundary condition was defined as the height, depth, and morphology of the alveolar bone [12]. The alveolar boundary condition in the cleft region was modified in more than 90% of the cleft children. This could be a reason for the indication of a CBCT examination before starting an orthodontic treatment or in cases that require tooth movement. Alveolar boundary conditions should be considered as a dynamic structure that may be changed by orthodontic treatment. A decrease in the buccal cortical bone thickness was noticed at the level of the second premolars by using active or passive self-ligating brackets [32]. The buccal and lingual cortical bone thickness may undergo changes in RME or slow palatal expansion treatment [33, 34]. In cleft children, the alveolar bone thickness is reduced in most cases, and the bone morphology is also changed. The secondary osteoplasty could also influence the anatomy in the dental frontal region. The mean age for surgical intervention is the age of eleven [35]. Therefore, a CBCT examination would be better indicated before the bone grafting surgery planning when it is useful for estimating the bone graft volume, but it could also reveal the cortical bone changes and 3D anatomy of the alveolar bone.

The jaw relationship could be assessed on CBCT images, but cephalometric measurements are also needed. In the present study, the cephalometric measurements and jaw relationship estimation were not reliable in 14% of children before eleven years of age due to the possible modifications induced by jawbone growth. However, jaw relationship changes were identified in 71.82% of the cases. The cephalometric analyses of the jaw relationships revealed most frequent in the cleft group the presence of maxillary hypoplasia with transversal and sagittal deficit of growth and Class III malocclusion with an anterior and lateral crossbite.

Maxillary asymmetry was frequently associated with a palatal cleft (87.5% of cases). The maxillary asymmetry needs to be corrected by rapid maxillary expansion and orthognathic surgery as well as soft tissue augmentation.

The prevalence of nasal cavity morphological changes in the studied group was 75% at the first CBCT examination in cleft children. It has been revealed that all complete anterior and total clefts show an alteration of nasal cavity morphology [36].

Regarding the treatment planning, the estimation of the bone graft volume on CBCT and planning of the graft type and fixation method were necessary before performing the bone grafting. There is a big controversy in the literature regarding the optimal timing for secondary osteoplasty. A systematic review concluded that the delayed hard palate repair had more positive effects on skeletal maxillary growth than the primary bone grafting before the age of three years [37]. After the age of ten, a bone reconstruction could also improve the canine eruption, and it is helpful for the orthodontic correction of the anterior jaw relationship [31].

The narrow maxilla was frequently associated with the palatal cleft [17]. Our study revealed that in 45.31% of cases, a maxillary transverse discrepancy was identified, but it could not be estimated before the age of eleven in 25% of cleft children. Orthodontic rapid maxillary expansion and slow maxillary expansion had similar results if the procedure was applied between eight to ten years of age. The changes after these procedures were observed in the nasal cavity width, maxillary width, and palatal cleft width in both the molar and premolar regions; and the alveolar crest width, arch width, tooth inclination, alveolar crest level, and buccal and lingual bone plate thickness only at the molar region [17].

Orthognathic surgery planning is based mainly on CBCT images. The recent guidelines in orthodontics considered unanimously that CBCT is justified if surgery is performed after the end of the jaw growth, around the age of twenty years [22]. In our study, the orthognathic surgery need was evaluated at different ages corresponding to the first age of examination of cleft children. The results showed that in more than 42.87% of cases, the CBCT could not predict the need for orthognathic surgery on CBCT and cephalometric measurements before twenty years of age.

### Limitations

The number of studied subjects was low, nevertheless the study identified the clinical situation of CLP patients under the age of ten in which CBCT could not foresee impaction or root resorption.

### Study strengths

Our study showed that the usefulness of CBCT scan in CLP patients varied with age, being more beneficial prior to the surgical planning of the bone grafting when it is useful for estimating alveolar bone morphology and changes of the cortical bone.

### Implications for practice and future research

The usefulness of CBCT examination in CLP patients should be further validated, explored, and expanded. Future research should also consider the cost-effectiveness of CBCT regarding the radiation dose according to the patient's age. The maxillofacial surgeon's and radiologist's experience should be consistent and documented in the literature.

## CONCLUSION

To be successful, the treatment must be based on adequate diagnosis in all stages. Correct evaluation and diagnosis of CLP cases rely undoubtedly on 3D imaging diagnosis. The timing for CBCT is very important to decrease the number of CBCT exposures and radiation doses in children. CBCT exposures before the age of eleven could not predict dental impaction, root resorption, or jaw relationships. The CBCT planning of the bone graft or the estimation of the transversal deficiency could also be performed after eleven years, and orthognathic surgery planning after twenty years. The postponement of the first CBCT examination in cleft children after the age of ten years when the alveolar bone grafting is also performed is recommended. To improve upon the limitations of the study, future studies should be undertaken.

## Data Availability

Further data is available from the corresponding author on reasonable request.
